# Revealing the Mg-Ion Storage Mechanism within a Covalent Organic Framework Electrode

**DOI:** 10.1021/acsaem.5c03247

**Published:** 2025-11-07

**Authors:** Matthew A. Wright, Alex R. Neale, Andrés Acín-Lalanza, Hui Gao, Matthew J. Rosseinsky, Andrew I. Cooper, Laurence J. Hardwick

**Affiliations:** † Department of Chemistry, University of Liverpool, Liverpool L697ZD, United Kingdom; ‡ Materials Innovation Factory, Liverpool L73NY, United Kingdom; § Stephenson Institute for Renewable Energy, Liverpool L69 7ZF, United Kingdom; ∥ The Faraday Institution, Harwell Campus, Didcot OX11 ORA, United Kingdom

**Keywords:** Covalent Organic Framework, *In Situ* Raman, Mg Battery, Battery Materials, Organic Electrodes, Raman Spectroscopy

## Abstract

Magnesium batteries offer a promising alternative to lithium-ion systems, but suitable electrodes remain limited. Covalent organic frameworks (COFs) are attractive candidates due to their structural tunability and open channels for ion transport. We report a pyrene- 4,5,9,10-tetraone COF composite with carbon nanotubes as a Mg electrode, delivering 70 mAh g^–1^ at 200 mA g^–1^ and operating at 1.3 V. *In situ* Raman spectroscopy confirms carbonyl-centered redox on pyrene tetraone, supporting a Mg^2+^-driven carbonyl reduction. Compared with Li^+^, only partial carbonyl utilization occurs, attributed to steric and electrostatic constraints of divalent Mg^2+^. This incomplete conversion to magnesium-enolate inspires future work toward structural optimization.

## Graphical abstract

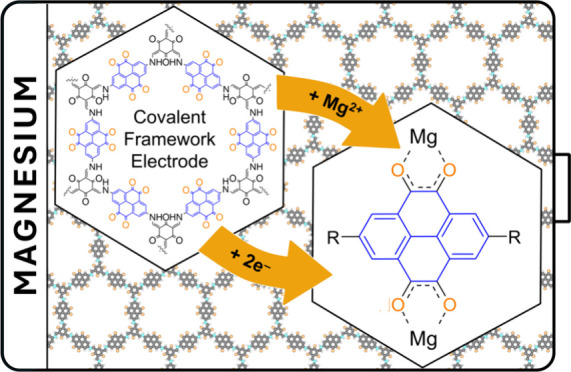

As battery use expands beyond portable consumer electronics, advanced energy storage technology will be crucial to meet the growing demand of twenty-first century.^1^ While lithium-ion batteries have dominated the market since their commercialization in 1991, efforts continue to find safer, cheaper and more sustainable alternatives. With lithium systems nearing their theoretical energy density,^2^ interest is shifting to other metals. Magnesium offers a promising option for grid-scale storage due to its low cost, low dendritic morphology, high abundance, and superior volumetric capacity (3833 mAh cm^–3^ for Mg vs. 2046 mAh cm^–3^ for Li and 1125 mAh cm^–3^ or Na).^3^

Although Li^+^ and Mg^2+^ have similar ionic radii (0.76 Å and 0.72 Å, respectively), most commercial lithium-ion battery materials perform poorly as hosts for magnesium ions.^4^ While several novel materials show promise, none yet meet all criteria for a viable Mg^2+^ electrode. For example, Mo_6_S_8_ displays reversibility toward Mg^2+^ insertion, achieving over 2000 cycles, but yields a low working potential of 1 V (vs. Mg^2+^/Mg);^5^ VS_4_ achieves high rate-performance but undergoes irreversible conversion reactions;^6^ and V_2_PS_10_ and V_2_Se_9_ offer fast Mg^2+^ diffusion and insertion-type mechanisms, but achieve low practical capacity.^7,8^ Conversion based electrodes like CuS, NiS and CoS_0.89_ can achieve high capacities but suffer from inferior cycling stability.^9,10^ Poor performances arise due to large electrostatic interactions in the hosts structure which struggles to accommodate and redistribute the incoming bivalent charge of Mg^2+^.

Alternatively, organic electrodes provide more flexible Mg^2+^ diffusion pathways with lower migration barriers, which can improve electrode kinetics and cyclability.^11^ Moreover, the structure of organic molecules can be easily modified, allowing for fine-tuning of electrochemical performances. To date, the use of small organic molecules has been limited by their solubility in organic electrolytes. However, polymeric materials have shown reversible magnesium (de)­insertion with solubility and reactivity tunable through monomer selection. Recent advances have demonstrated high-energy-density in organic electrodes, particularly those containing carbonyl C=O groups, achieving highly reversible Mg^2+^ insertion at high potentials.^12^ Poor electronic conductivity of polymer electrodes remains a challenge.^13^

Covalent organic frameworks (COFs) present a promising new research direction for organic electrode materials. COFs possess extended crystalline structures comprising discrete organic molecules as structural building blocks which are connected by covalent bonds.^14^ Unlike small organic molecules, they are typically insoluble in organic solvents making them well suited to battery applications.^15^ Their ordered frameworks provides highly porous structures with uniform pore sizes, which is well suited to Mg-ion diffusion,^16^ and allows for high utilization of redox-active sites.^17^ Similar to polymers, their modular structure provides a high degree of tunability, as functional groups can be introduced in controllable and predictable locations.^18^ As highlighted in a recent perspective, COFs serve as model systems for fundamental and mechanistic understanding of organic battery design principles.^19^ However, despite their potential, COFs are still largely unexplored as electrode materials for magnesium batteries, with only a limited number of examples reported to date. Pseudocapacitive Mg^2+^ storage has been so far been demonstrated in triazine-,^20^ pyrazine-^21^ and quinone-based^22^ COFs, where coordinated redox reactions at C=O and C=N sites enable good cycling stability and working potentials near 1.3 V (vs. Mg^2+^/Mg). However, recent work on anthraquinone-based COFs demonstrates that electrolyte and binder selection can strongly affect capacity and cycling stability; with LiTFSI in ethereal solvents and polytetrafluoroethylene binder delivering near-theoretical capacity and excellent long-term stability, while carbonate electrolytes and polyvinylidene fluoride binder led to poor performance.^23^ While π-π stacking offers some electrical conductivity,^24^ composite electrodes containing conductive carbons are often needed for effective electrochemistry.^25^ Additionally, chloride-containing electrolytes have been shown to hinder reversible Mg^2+^ storage, instead requiring chloride-free systems such as Mg­[TFSI]_2_ or aqueous MgSO_4_.^20,23^

Previously, we have reported high-capacity toward Li-ion insertion in COF/carbon nanotube (CNT) composites utilizing redox active C=O moieties on phenanthrene (tricyclic) and pyrene (tetracyclic) rings with triformylphloroglucinol (TFG) linkers.^26,27^ The optimized composite of pyrene-4,5,9,10-tetraone COF (containing 50 *wt*% CNT, PT-COF-50) delivered a record-high specific capacity of 280 mAh g^–1^ at 200 mA g^–1^ (normalized to the COF material) and exhibited excellent rate performance, retaining 82% of its capacity at 5000 mA g^–1^. *In situ* Raman microscopy and electrochemical studies revealed a reversible 4-electron/4-Li^+^ redox mechanism with 95–98% redox-active-site utilization of the pyrene carbonyl groups.

Here, we investigate PT-COF-50 for use as an electrode in secondary Mg^2+^ cells, demonstrating reversible insertion of Mg^2+^. *In situ* Raman spectroscopy reveals a redox mechanism centered on the carbonyl groups of the pyrene tetraone units, with characteristic C=O and C=C band shifts supporting carbonyl reduction during incorporation of Mg^2+^ ions. PT-COF-50 was synthesized via imine condensation of 2,7-diaminopyrene-4,5,9,10-tetraone (DAPT) and triformylphloroglucinol (TFG), forming β-ketoenamine linkages, in the presence of CNTs (50 *wt*%) [Figure 1] in mesitylene, 1,4-dioxane and acetic acid. The resulting precipitate was filtered and washed by Soxhlet extraction and dried under vacuum before being processed into electrodes for electrochemical characterization. Detailed descriptions of the synthesis protocols well as comprehensive characterization have been reported in earlier work and are provided in the Supporting Information.^26,27^ The capacity of pure CNT was determined previous to be 13 mAh g^–1^ albeit in a lithium-ion containing electrolyte and we discuss the capacity contribution from CNT in the composite in the SI. An electrolyte containing 0.5 M magnesium bis­(trifluoromethylsulfonyl)­imide (Mg­[TFSI]_2_) in dimethoxyethane (DME)/1-methoxy-2-propylamine (MPA) (3.84:1, *w*/*w*) was selected for electrochemical characterization of PT-COF-50. The added amine MPA acts as a Lewis base and strongly coordinates to Mg^2+^, helping to dissociate Mg^2+^ from TFSI^–^ anions, promoting higher ionic conductivity and enabling more reversible Mg plating and stripping at the metallic Mg anode.^28^ Moreover, Mg­[TFSI]_2_ based electrolytes do not contain Cl^–^ species (unlike traditional Grignard-based electrolyte systems for Mg batteries), which is known to corrode cell components,^29^ form complex Mg_*x*_Cl_*y*_ species which disrupt Mg^2+^ insertion^+^,^30^ and reduce the performance of COF electrodes.^23^ An open circuit potential (OCV) of approximately 1.7 V vs. Mg^2+^/Mg was recorded over 36 h after cell assembly, indicating that PT-COF-50 does not undergo any self-discharge in 0.5 M Mg­[TFSI]_2_/DME/MPA electrolyte [Figure S4]. Electrochemical (de)­insertion of Mg^2+^ was performed between 0.8 and 2.8 V vs. Mg^2+^/Mg to allow for direct comparison with previously reported cycling of against lithium (equivalent to 1.5–3.5 V vs Li^+^/Li),^27^ without risk of electrolyte oxidation [Figure S5].

**Figure 1:**
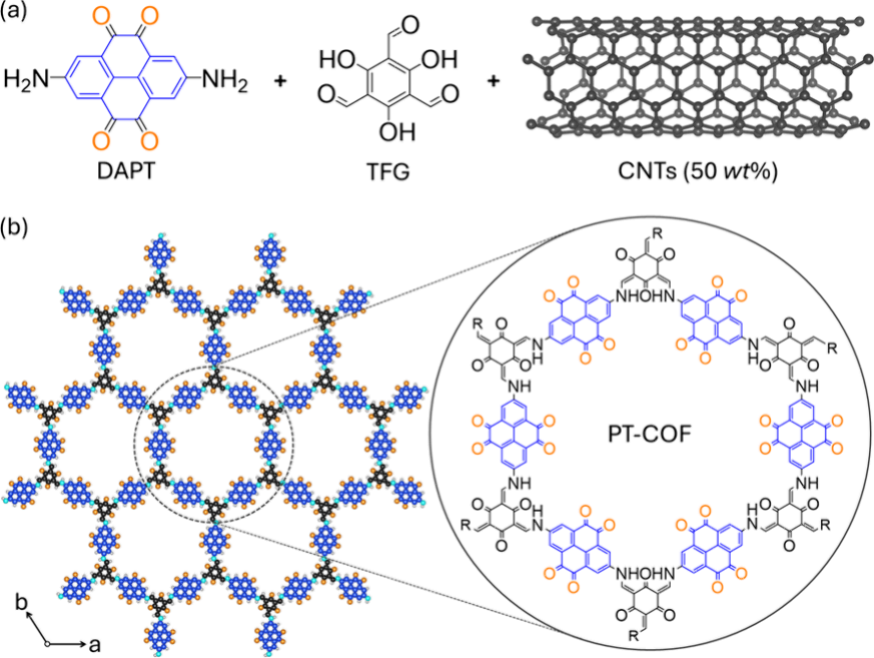
(a) The structures 2,7-diaminopyrene-4,5,9,10-tetraone (DAPT) of triformylphloroglucinol (TFG) monomers and a schematic representation of carbon nanotubes (CNTs). (b) The extended crystalline structure of PT-COF. A skeletal representation of the structure is shown in the inset circle. The aromatic pyrene unit is colored blue, and the tetraone carbonyl oxygens are colored orange.

Cyclic voltammetry (CV) was performed at a sweep rate of 0.5 mV s^–1^ for multiple cycles showing the redox chemistry of PT-COF-50 with the Mg^2+^-based electrolyte [Figure 2a]. While pseudocapacitive storage mechanisms have been previously observed in other COF materials containing β-ketoenamine linkages,^31,32^ the cyclic voltammetry shown here is more consistent with faradaic, redox chemistry, rather than surface-confined capacitive behavior. The first cycle shows an onset of reduction almost immediately as the potential decreases below the OCV. From cycle 2 onward, the onset of reduction occurs at *ca*. 2.35 V vs. Mg^2+^/Mg succeeded by a primary, broad reduction peak at 1.6 V vs. Mg^2+^/Mg. During the reverse process, oxidation reactions proceed above 1.6 V with a primary peak at *ca*. 2.2 V and several minor peak features at higher voltages. This differs from the electrochemical response observed for this material when cycled against metallic lithium in Li^+^-based electrolytes, where two primary peaks are observed that each split individually to indicate four 1-electron processes (occurring at 2.9, 2.7 2.3, 2.1 V vs. Li^+^/Li in the reductive direction) which are ascribed to the reduction of the four carbonyl groups in PT-COF-50.^27^ Herein, one broad reductive peak (possibly comprising two overlapping responses) in Mg^2+^-electrolyte indicates either no distinct transitions are detectable at this scan rate, or the complete reduction of the 1,2-diketone group does not occur within this potential range (which is explored later). At potentials above 2.75 V, the oxidative current is attributed to slight oxidation of the electrolyte solution [Figure S2]. Nonetheless, multiple CV curves maintain similar profiles; with comparable peak current densities and integral charge, suggesting good reversibility of the electrochemical processes occurring within the cell over this potential range [Figures S5–S6].

**Figure 2:**
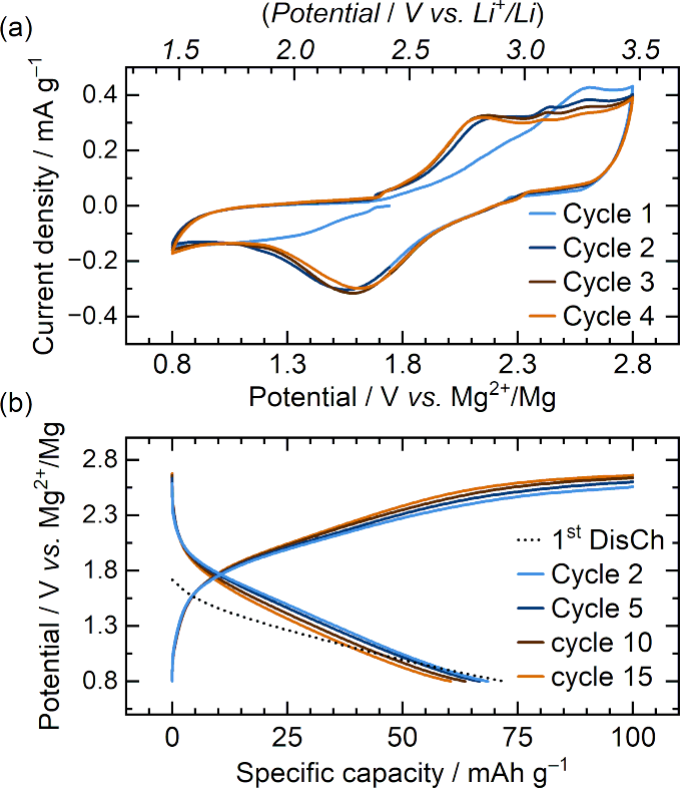
Electrochemical characterization of PT-COF-50 in 0.5 M Mg­[TFSI]_2_/dimethoxyethane/1-methoxy-2-propylamine (3.84:1, *w/w*). (a) Cyclic voltammetry was performed between potential limits of 0.8–2.8 V vs. Mg^2+^/Mg at a sweep rate of 0.5 mV s^–1^. Conversion between potential (V) against Mg^2+^/Mg and Li^+^/Li is given on the top *x*-axis of panel (a). (b) Galvanostatic charge–discharge was performed at a current density of 200 mA g^–1^, and data were recorded at 5 mV increments. The first discharge (from an open circuit potential of 1.7 V is shown by the dashed black line). A charge-capacity cutoff of 100 mAh g^–1^ was applied to limit capacitive contribution of electrolyte oxidation above 2.7 V.

Galvanostatic charge–discharge was performed at a current density of 200 mA g^–1^ between 0.8 and 2.8 V vs Mg^2+^/Mg [Figure 2b]. Similarly to CV data, the first discharge process displays a sloping plateau almost immediately upon Mg^2+^ insertion and achieves a discharge capacity of 72 mAh g^–1^ (normalized to the COF material). A high-potential plateau is observed (around 2.75 V) during charging which relates to electrolyte oxidation. Charge capacity was therefore limited to 100 mAh g^–1^ to minimize electrolyte decomposition with each cycle, Subsequent cycles maintain similar sloping load curves to with a higher average discharge potential of 1.3 V vs Mg^2+^/Mg and a discharge capacity of 60 mAh g^–1^ is maintained after 15 cycles, yielding Coulombic efficiencies of 60–70%. The electrochemical performance of PT-COF-50 is comparable with other anthraquinone-based COFs which achieve reversible capacities of 50 mAh g^–1^ at 200 mA g^–1^, achieved through partial enolate formation yielding average an potential of 1.2 V in chloride-free electrolytes.^23^ It is also comparable with triazine-based COFs which achieve 70 mAh g^–1^ at 200 mA g^–1^ and average potentials of 1.3 V via reversible redox at the C=N site in chloride-free electrolytes.^20^ While these potentials are reasonably high compared to inorganic Mg-ion insertion electrodes that tend to have an average potential in the region of 1.0–1.5 V,^3,5,33^ they are lower than the equivalent potential achieved for the same material when cycled against lithium (2.55 V vs Li^+^/Li which equates to 1.87 V vs Mg^2+^/Mg).^27^ This decrease in potential may be attributed to stronger Coulombic interactions between Mg^2+^ and the host structure as the high charge-per-size Mg^2+^ binds more strongly to the electrodes active sites than Li^+^, resulting in high bulk transport barriers and requiring large overpotentials. Similar differences in performance between Mg^+^ cells and Li^+^ cells are observed in other anthraquinone-based COFs.^23^ Higher capacities and working potentials may be achieved in chloride containing electrolytes, however *ex situ* infrared and energy-dispersive X-ray spectroscopy have revealed a mechanism dominated by magnesium-chloride complexes, rather than uncoordinated Mg^2+^, coordinating to reduced carbonyls.^23^ These Mg–Cl complexes act like monovalent, solvated cationic species, allowing for a reduced local electrostatic penalty for electron uptake at the carbonyl. For electrode design this regime is less useful because it masks the intrinsic challenges of divalent Mg^2+^ storage, introduces corrosive/instability pathways tied to chloride chemistry, and does not provide a transferable route to frameworks that will perform under chloride-free, practical Mg electrolytes.

To follow the mechanism of Mg^2+^ storage in PT-COF based electrodes and make comparisons with the carbonyl-based COF electrochemistry with the lithium-based system, *in situ* Raman microscopy was used to study the material during electrochemical Mg^2+^ insertion. While the CNTs in the active material provide clear electrochemical advantages due to the intimate growth of the COF as a *shell* on the conductive CNT structure, a free-standing electrode using PT-COF synthesized without CNTs (rather than PT-COF-50) was used for spectroscopic measurements. This avoids challenges relating to large dominating carbon (CNT) vibrational bands in the Raman spectra while retaining a crystalline COF active material.^27^ Figure 3(a) shows the evolution of the main Raman bands during linear sweep voltammetry from 2.6 to 0.8 V vs. Mg^2+^/Mg against a Mg-metal counter electrode. Figure 3(b) comprises selected Raman spectra duplicated from Figure 3(a) to highlight the main changes in the measured spectra overlaid with the spectrum of the pristine, dry PT-COF electrode. Therein, spectral intensities are normalized with respect to the major band at *ca*. 1600 cm^–1^ to facilitate straightforward relative comparisons.

**Figure 3:**
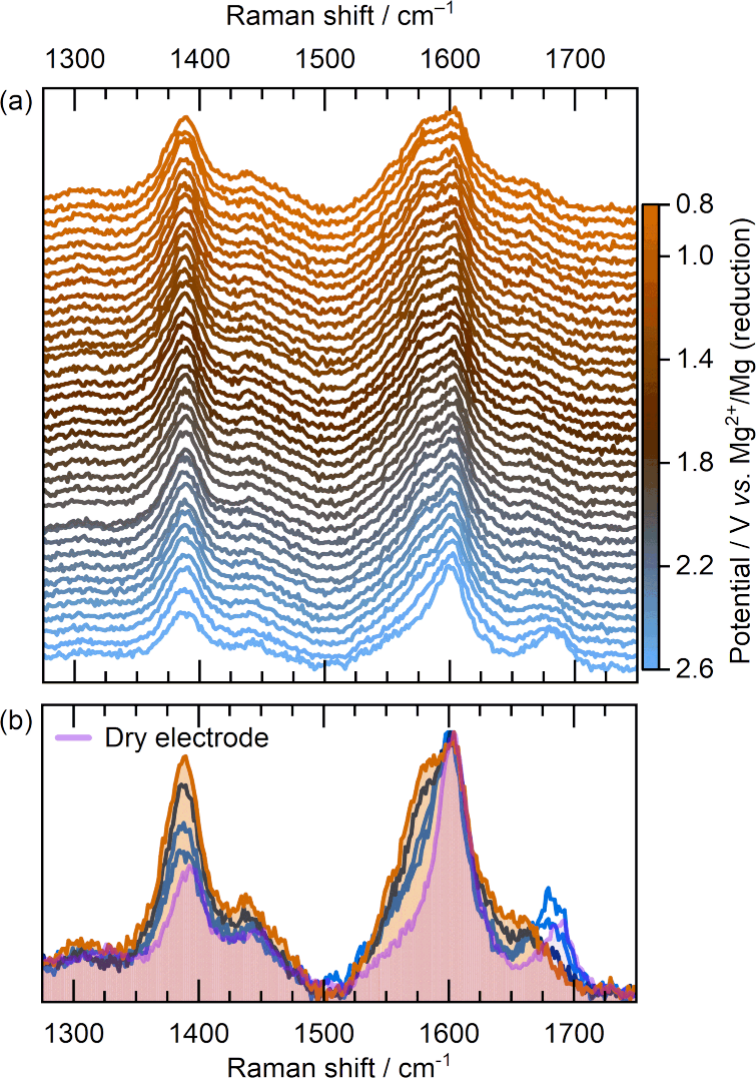
(a) *In situ* electrochemical Raman microscopy spectra of a free-standing PT-COF electrode during reduction in 0.5 M Mg­[TFSI]_2_/dimethoxyethane/1-methoxy-2-propylamine (3.84:1, *w/w*) electrolyte during linear sweep voltammetry from 2.6 to 0.8 V vs Mg^2+^/Mg at 0.3 mV s^–1^. (b) Selected Raman spectra duplicated from the top panel using the equivalent colormap overlaid with the Raman spectrum of the dry PT-COF electrode. The colormap scale shows the cell potential (*E*) vs. Mg^2+^/Mg for the presented spectrum, and the shaded areas in (b) highlight the broad differences between pristine PT-COF and the electrode after full Mg insertion. All spectra were collected between 300 and 2000 cm^–1^ [Figure S7] and are baseline subtracted and normalized with respect to the primary band intensity at *ca*. 1600 cm^–1^.

The primary changes in the measured spectra support the electrochemical reduction of carbonyl chemistry on the pyrene-4,5,9,10-tetraone group, relating to the C=O stretching mode at ca. 1680–1690 cm^–1^ and a band/shoulder formation/shift at ca. 1570–1580 cm^–1^. Both primary regions are affected by ion-dipole interactions upon wetting of the electrode with the Mg^2+^-based electrolyte [Figure 3b] with, most notably, the C=O stretching mode broadening and shifting to lower wavenumbers. This is analogous to the band shifts observed upon wetting in the Li^+^-based electrolyte.^27^

Upon electrochemical magnesium insertion, the relative C=O band intensity reduces, broadening with the formation of a weak secondary band at *ca*. 1660 cm^–1^ attributed to the transition toward the enolate (C–O–Mg) bond character. This transition coincides with the formation and growth of the band at *ca*. 1570–1580 cm^–1^ relating to changing bond structure of the C–C/C=O bond from the functional 1,2-diketone group. Herein, comparison with the monovalent Li^+^-based electrochemistry of PT-COF provides a useful indicator for the depth of complete carbonyl reduction.^27^ Figure S8 shows the Raman spectra of PT-COF after complete reduction in Li^+^ and Mg^2+^-based electrolytes, highlighting more pronounced differences in Li^+^-based electrochemistry ascribed to complete formation of the lithium enolate and the associated C=C double bond that links both carbonyl/semienolate functionalities. The measured capacity in Li^+^-based electrolyte, combined with the pronounced and obvious Raman shifts, supports the complete utilization of all carbonyl active sites (i.e., 2 electrons per 1,2-diketone group). Comparatively in the Mg^2+^ electrolyte, the shifts are considerably less pronounced. In agreement with the lower measured electrochemical capacity and the fewer peaks measured by voltammetry [Figure 2], the less distinct band growth and shifts in the Raman spectra are indicative of only partial reduction in the presence of the bulkier divalent cation.

The partial utilization of carbonyl groups is further supported by the behavior of the band at 1390 cm^–1^ In Li^+^ electrolyte, this band shifts progressively to lower wavenumbers [Figure S8] particularly during the second 2 electron reduction step because of increasing aromaticity in the pyrene rings when the two C=C double bonds form on opposite faces of the fused ring structure. However, in this work, while the relative band intensity grows during magnesium insertion, no obvious shifts in the band position at 1390 cm^–1^ are observed, suggesting aromaticity in the fused rings is less affected. Overall, these results confirm the activity of carbonyl electrochemistry with the divalent Mg^2+^ cation, undergoing reduction of the carbonyl group during discharge, but point toward incomplete utilization of the carbonyl redox active sites within the PT-COF channels without the complete formation of the magnesium enolates (and the associated C=C linker) that was observed in Li^+^ electrolyte. This limitation may arise due to electrostatic limitations within the channels possibly inhibiting the second electron reduction for each 1,2-diketone group (or this requiring significantly more negative potentials) and/or by the channel being too narrow (1.8 nm) to host sufficient counter-cations to balance the reduction of 12 total carbonyl groups per COF ring: i.e., ranging from 6 Mg^2+^ cations in perfect bidentate coordination structures to 12 [Mg­(solvent)_*x*_[TFSI]_1_]^+^ solvated contact-ion pair species.

As outlined in the introduction, one benefit of COF electrodes is the high degree of chemical and structural tunability. The modest capacity observed for PT-COF-50 is attributed to incomplete redox site utilization, with some of the sites used by Li^+^ not accessed by Mg^2+^. This inspires further investigation into structural optimization of framework structures to facilitate higher-degrees of Mg–O coordination, either by changing the location and geometry of carbonyl groups or by tuning the pore size by selecting different sized quinone monomer units (as has been previously explored for Li^+^ insertion into benzoquinone, naphthoquinone and anthraquinone-based electrodes) to increase capacity, and achieve higher energy densities.^23^ Additionally, redox reactions may be altered by replacing carbonyl groups with other redox-active moieties, such as imines or thiols, or by incorporating heteroatoms into the aromatic carbon backbone, For example, experimental studies supported by density functional theory (DFT) have shown that lone pair electrons on nitrogen atoms in triazine units can participate in dipole–quadrupole interactions, enhancing both capacity and cycling stability.^20,34^ Advancements in this direction may enable the rational design of next-generation electrode materials not only for Mg^2+^ storage but also for other earth-abundant multivalent ions such as Ca^2+^ and Al^3+^.

In summary, reversible Mg^2+^ insertion has been demonstrated in PT-COF-50 composites with CNTs as organic electrodes for Mg-ion batteries. *In situ* Raman microscopy confirmed the electrochemical activity of the pyrene-4,5,9,10-tetraone groups is attributed to the redox activity of the carbonyl groups in the presence of divalent Mg^2+^. These results show that COFs can provide efficient Mg^2+^ diffusion pathways and offer a real alternative to inorganic Mg electrode materials. However, both electrochemical and Raman spectroscopic characterization point toward an incomplete utilization of the available carbonyl functionalities within PT-COF, especially compared with Li^+^ electrolytes. Likewise, while comparable operational potentials to similar Mg-electrodes is achieved, potentials were low relative to contemporary Li^+^ ion systems. This inspires future work to optimize structural and chemical properties by leveraging the highly tunable nature of COFs to incorporate more accessible redox active sites and opens the door to other multivalent cations, providing a clear route to the design of higher energy density electrodes for post-lithium batteries.

## Supplementary Material



## Notes

Additional experimental details, materials and methods; electrolyte preparation and testing; additional electrochemical testing; and further Raman spectroscopy (PDF)

## ⊥

Materials Research Laboratory, University of California, Santa Barbara, California 93106, United States

## #

School of Chemistry and Chemical Engineering, Huazhong University of Science and Technology, Wuhan 430074, China.
