# Supplementary material for: Revealing the Mg-Ion Storage Mechanism within a Covalent Organic Framework Electrode
Source: ACS Appl Energy Mater. 2025 Nov 7;8(22):16451–6. doi: 10.1021/acsaem.5c03247 (PMC12648465; doi:10.1021/acsaem.5c03247)
Supplement: Supplementary file 1 [file ae5c03247_si_001.pdf]

Revealing the Mg-Ion Storage Mechanism Within a Covalent Organic Framework Electrode

Matthew A. Wright[a,b,c,d,†], Alex R. Neale[a,c,d], Andrés Acín-Lalanza[a,b,c], Hui Gao[a,c,‡], Matthew J. Rosseinsky[a,b], Andrew I. Cooper[a,b], Laurence J. Hardwick[a,c,d*]

[a] Department of Chemistry, University of Liverpool, Liverpool, L69 7ZD, United Kingdom
[b] Materials Innovation Factory, Liverpool, L7 3NY, United Kingdom
[c] Stephenson Institute for Renewable Energy, Liverpool L69 7ZF, United Kingdom
[d] The Faraday Institution, Harwell Campus, Didcot, OX11 ORA, United Kingdom
† Current Address: Materials Research Laboratory, University of California, Santa Barbara, CA 93106, United States
‡ Current Address: School of Chemistry and Chemical Engineering, Huazhong University of Science and Technology, Wuhan 430074, China
* Corresponding Author: hardwick@liverpool.ac.uk

Experimental information

PT-COFX synthesis – Triformylphloroglucinol (TFG) were purchased from Tokyo Chemical Industry UK Ltd. The synthesis of 2,7-diaminopyrene-4,5,9,10-tetraone (DAPT) has been explained in detail previously.[1–3] Carbon nanotubes (CNTs) were 7-15 nm in outer diameter and 0.5-10 μm in length and were sourced from Sigma Aldrich. 10.5 mg (0.05mmol) TFG, 21.9 g (0.075 mmol) DAPQ, CNT (50 *wt*% of the composite based on the yield of PT-COF), 0.9 mL mesitylene (Acros Organics), 0.3 mL 1,4-dioxane (Alfa Aesar) and 0.2 mL of 6 M acetic acid (Sigma Aldrich) was added to a 10 mL Pyrex tube and homogenized by sonication for 30 minutes before degassing with three cycles of LN2 assisted freeze-pump-thaw and left evacuated to an internal pressure of 100 mTorr before hermetically sealing and heating in an oven to 120 °C for 72 hours. The product (in the form of a black precipitate) was collected by filtration and washed with DMF, DMSO and finally acetone before drying in a desiccator. Any remaining guest molecules within the structure were removed by Soxhlet extraction with methanol for 24 hours. The final powder was dried under vacuum at 85 °C.

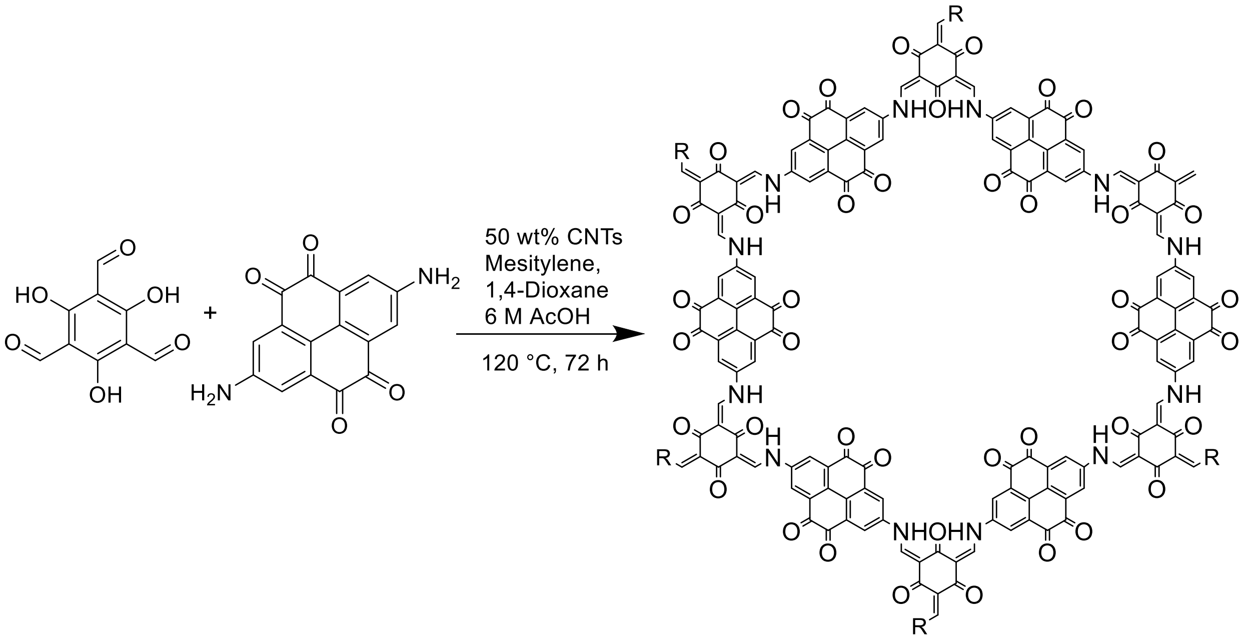

Figure S1. Reaction scheme for the synthesis of PT-COF-50 Triformylphloroglucinol and 2,7-diaminopyrene-4,5,9,10-tetraone in the presence of carbon nanotubes.

Electrolyte preparation – The electrolyte consisted of a 0.5 M solution of magnesium bis(trifluoromethane)sulfonimide ($Mg(TFSI)_2$, 99.5 %, Solvonic) dissolved in a mixture of dimethoxyethane (DME, Sigma Aldrich, anhydrous, inhibitor free, 99.5 %) and 1-methoxypropyl-2-amine (MPA Sigma Aldrich, 99 %) in a 3.84/1 *w/w* ratio.[4] $Mg(TFSI)_2$ was dried at 220 ˚C under a dynamic vacuum of ≈5 mbar for 12 h. DME was further dried over activated molecular sieves (Sigma Aldrich, 3 Å, heated to 250 °C under vacuum) to a H_2O content of < 10 ppm by Karl-Fischer titration. MPA was degassed by freeze-pump-thaw with liquid N_2 and dried by distillation over CaH_2 before storing over activated molecular sieves. A final water content of < 10 ppm was given by Karl-Fischer titration. Electrolyte stability was confirmed by cyclic voltammetry at 10 mV s^{-1} between –1.0-3.0 V vs Mg^{2+}/Mg to perform Mg plating and stripping using a carbon-coated Al current collector foil as the working electrode [**Figure S2**], and galvanostatic cycling at a current density of 0.5 mA cm^{-2} in a Mg-Mg symmetric cell [**Figure S3**].

PT-COF-50 electrode preparation – The PT-COF-50 active material was first ground with carbon black (Super C65, Imerys) by mortar and pestle and then dispersed with poly(vinylidene fluoride) (PVDF)binder in N-methylpyrrolidone (NMP). The slurries were mixed using a planetary centrifugal mixer (Thinky ARE-250, Intertronics) to produce a slurry with mass ratios of 7:2:1 (PT-COF-50:Carbon black:PVDF). The resulting well dispersed slurry was cast onto carbon-coated Al current collector foil by doctor blade. After initial drying to remove the bulk of the NMP solvent, electrode discs (12 mm diameter) were punched and subsequently dried under vacuum at 80 °C and transferred directly to an Ar filled glovebox (H_2O, O_2 < 0.1 ppm).

Electrochemical measurements – Electrochemical characterisation was performed on stainless steel coin cells (CR2032, Pi-KEM) assembled under inert Ar atmosphere inside a glovebox (MBraun LabMaster Pro-Eco) with O_2 and H_2O levels < 0.1 ppm. Negative electrodes consisted of polished Mg foil discs with a diameter of 16 mm. Electrodes were separated by Whatman GF/D borosilicate glass fibre membranes soaked with 0.5 M $Mg(TFSI)_2$/DME-MPA electrolyte to a total volume that can be estimated to be 500-750 μL. Electrochemical measurements were conducted using a BioLogic VSP300 potentiostat with cells maintained at 30 ˚C. Galvanostatic charge-discharge was performed between potential limits of 0.8-2.8 V *vs*. Mg^{2+}/Mg at a current density 200 mA g^{-1}, recording the change in potential every 5 mV. Cyclic voltammetry was recorded between potential limits of 0.8-2.8 V at a sweep rate of 0.5 mV s^{-1}.

Capacity contribution of CNT in the PT-COF-50 composite – The specific capacities of the PT-COF-50 composites were calculated based on the mass of the PT-COF in the composite, however both PT-COF and CNT contribute to the overall capacity of the electrode. The specific capacity of pure CNT is 13 mAh g^{-1} (as we have reported previously in a lithium containing electrolyte).[1] The capacity contribution CNT in the PT-COF-50 composite is calculated using equation S1:

S1:

$$C_{\mathrm{PT-COF-50}} - C_{\mathrm{PT-COF}} = \frac{b \times C_{CNT}}{a}$$

Where a and b are the relative contents of PT-COF and CNT.

***In situ* Raman microscopy –** The free-standing working electrode, comprising PT-COF, conductive carbon (Super C65), and poly(vinylidene fluoride-hexafluoropropylene) co-polymeric binder (Kynar-flex, Arkema) binder, was prepared as described previously. In short, the PT-COF, carbon, and binder were dispersed along with dibutyl phthalate (DBP, as a plasticizer) at ratios of 4:1:2:3 in acetone. After mixing, the slurry was cast on to a glass tile using a doctor blade (60 μm). After the acetone evaporated, electrode discs (8 mm) were punched from the film and washed with diethyl ether to extract the DBP. The resulting free-standing electrodes were then dried under vacuum at 80 °C and transferred directly into the Ar filled glovebox (H_2O, O_2 < 0.1 ppm).

The *in situ* Raman cell (ECC-Opto-Std, El-Cell) was prepared and sealed under the Ar atmosphere. A polished disc of Mg was used as the counter/reference electrode and a glass microfibre (Whatman GF/F) was used as the separator. The separator was wetted with the electrolyte 0.5 M $Mg[TFSI]_2$ in dimethoxyethane/1-methoxy-2-propylamine (3.84:1, w/w). Lastly, the free-standing PT-COF electrode was

pressed against a carbon-coated aluminium foil current collector with a small hole (3 mm) punched at the centre for optical access to the rear of the free. The cell was hermetically sealed with a CaF_2 optical window before being rested for ca. 20 h to allow full wetting of the electrode and then transferred to the spectrometer.

Raman spectra were collected using a Renishaw In-Via Raman spectrometer equipped with an upright microscope and a 50x objective. A 532 nm excitation laser wavelength was used at a power of *ca*. 0.027 mW, with 3 x 45 s accumulations per spectrum. The working electrode was first swept from OCP (*ca*. 2 V *vs.* Mg^{2+}/Mg) up to 2.6 V *vs*. Mg^{2+}/Mg and held for 20 mins. Spectra were then collected during the negative going sweep at 0.3 mV s^{-1} down to 0.8 V *vs*. Mg^{2+}/Mg. One spectrum was collected every 3 mins equating to *ca*. one spectrum per 54 mV.

Supporting data

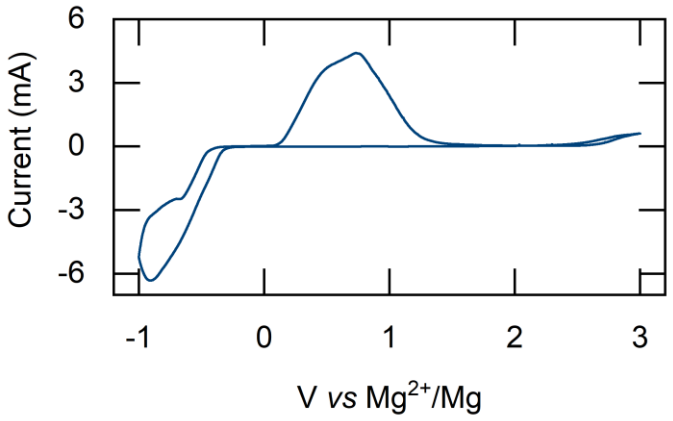

Figure S2. The second cycle CV curve for Mg plating and stripping at a sweep rate of 10 mV s^{-1} in 0.5 M $Mg(TFSI)_2$/DME-MPA between –1.0-3.0 V *vs* Mg^{2+}/Mg using a carbon-coated Al current collector foil as the working electrode.

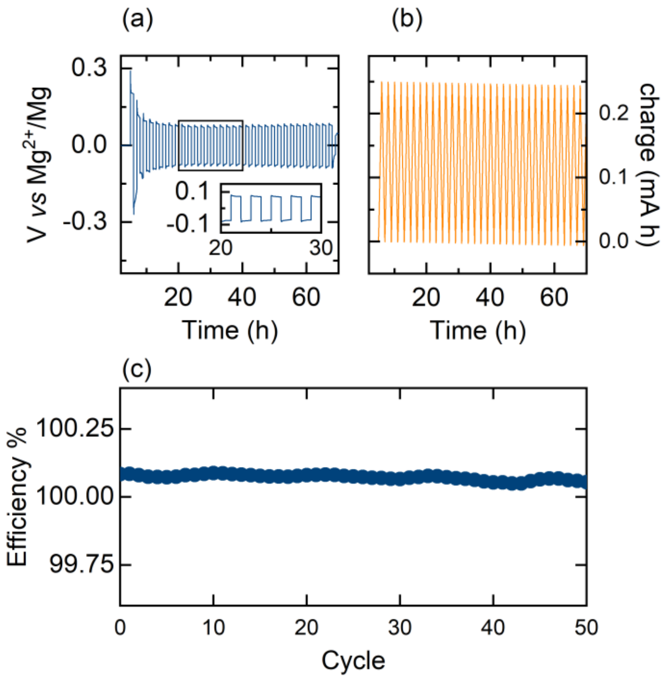

Figure S3. Galvanostatic cycling for a Mg-Mg symmetric cell containing 0.5 M $Mg(TFSI)_2$/DME-MPA at a current density of 0.5 mA cm^{-2}. Panels show (a) potential *vs* time, (b) charge *vs* time, and (c) coulombic efficiency over 50 cycles.

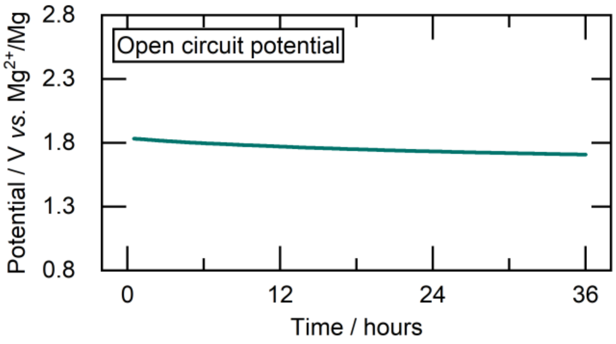

Figure S4. The open circuit potential measured at 30 °C over 36 hours after cell assembly. Cells were allowed to rest for 36 hours, to allow the electrolyte to properly permeate and interface with the separator and electrode surfaces, achieving a stable open circuit potential of approximately 1.7 V.

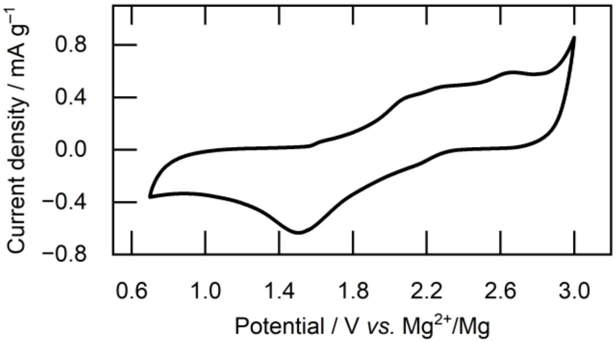

Figure S5. Cyclic voltammetry of PT-COF-50 at a sweep rate of 5 mV s^{-1} between 0.7–3.0 V vs Mg^{2+}/Mg. At potentials beyond 2.77 V there is a spike in oxidative current indicating the oxidative stability of the electrolyte solution.[4]

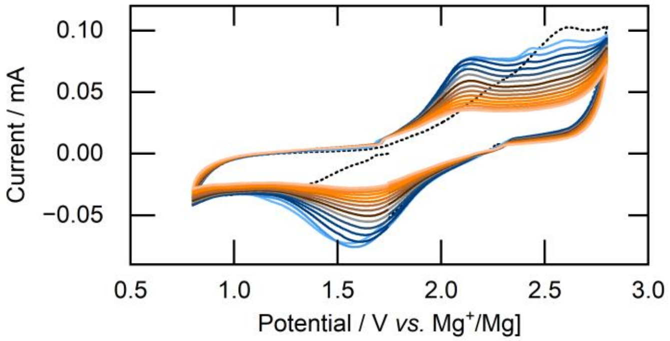

Figure S6. Cyclic voltammetry of PT-COF-50 at a sweep rate of 5 mV s^{-1} between cycles 1 (dotted black line) and 16 (orange line).

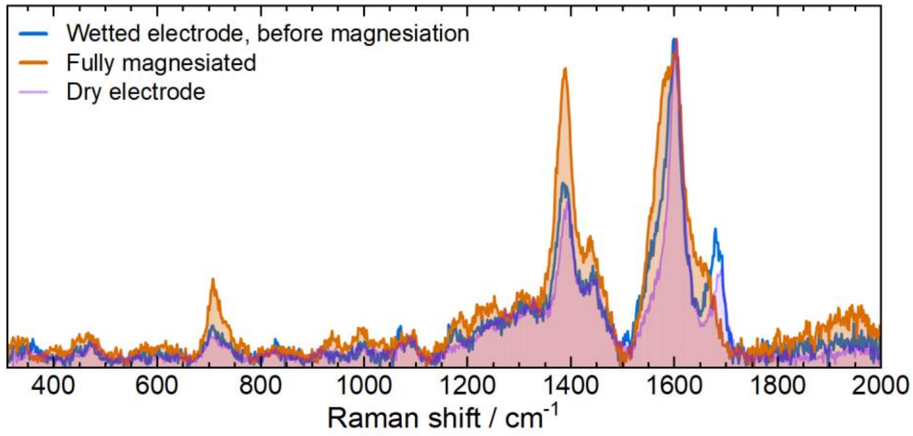

Figure S7. *In situ* electrochemical Raman microscopy spectra of a free-standing PT-COF electrode during reduction in 0.5 M Mg[TFSI]$_2$/dimethoxyethane/1-methoxy-2-propylamine (3.84:1, *w/w*) electrolyte. All spectra were collected between 300-2000 cm^{-1} and are baseline subtracted and normalized with respect to the primary band intensity at *ca*. 1600 cm^{-1}.

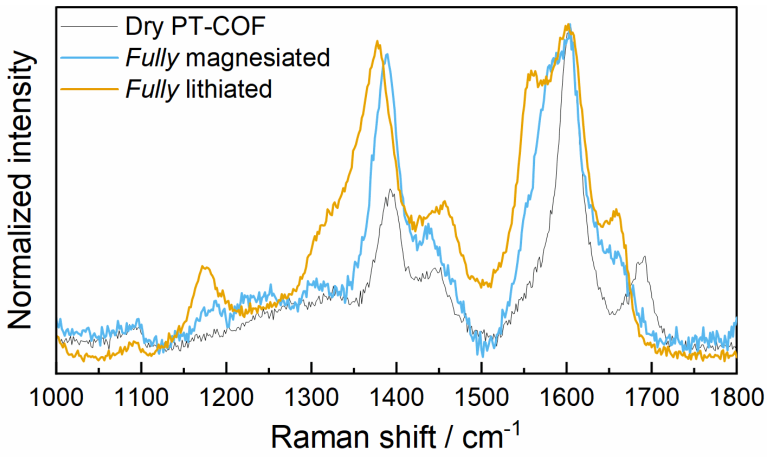

Figure S8. Comparison of Raman spectra of PT-COF electrode following electrochemical reduction in Mg^{2+}-based (blue) and Li^{+}-based (orange) electrolytes compared with the pristine, dry PT-COF electrode (grey).

References

(1) Gao, H.; Neale, A. R.; Zhu, Q.; Bahri, M.; Wang, X.; Yang, H.; Xu, Y.; Clowes, R.; Browning, N. D.; Little, M. A.; Hardwick, L. J.; Cooper, A. I. A Pyrene-4,5,9,10-Tetraone-Based Covalent Organic Framework Delivers High Specific Capacity as a Li-Ion Positive Electrode. *J. Am. Chem. Soc.* **2022**, *144* (21), 9434–9442. https://doi.org/10.1021/jacs.2c02196.

(2) Letizia, J. A.; Cronin, S.; Ortiz, R. P.; Facchetti, A.; Ratner, M. A.; Marks, T. J. Phenacyl–Thiophene and Quinone Semiconductors Designed for Solution Processability and Air-Stability in High Mobility n-Channel Field-Effect Transistors. *Chem. – Eur. J.* **2010**, *16* (6), 1911–1928. https://doi.org/10.1002/chem.200901513.

(3) Xing, Q.; Song, K.; Liang, T.; Liu, Q.; Sun, W.-H.; Redshaw, C. Synthesis, Characterization and Ethylene Polymerization Behaviour of Binuclear Nickel Halides Bearing 4,5,9,10-Tetra(Arylimino)Pyrenylidenes. *Dalton Trans.* **2014**, *43* (21), 7830–7837. https://doi.org/10.1039/C4DT00503A.

(4) Hou, S.; Ji, X.; Gaskell, K.; Wang, P.-F.; Wang, L.; Xu, J.; Sun, R.; Borodin, O.; Wang, C. Solvation Sheath Reorganization Enables Divalent Metal Batteries with Fast Interfacial Charge Transfer Kinetics. *Science* **2021**, *374* (6564), 172–178. https://doi.org/10.1126/science.abg3954.
